# Fast urinary screening of oligosaccharidoses by MALDI-TOF/TOF mass spectrometry

**DOI:** 10.1186/1750-1172-9-19

**Published:** 2014-02-06

**Authors:** Laurent Bonesso, Monique Piraud, Céline Caruba, Emmanuel Van Obberghen, Raymond Mengual, Charlotte Hinault

**Affiliations:** 1 Biochemistry Laboratory, University Hospital, Nice, France; 2 University of Nice-Sophia Antipolis, Institute for Research on Cancer and Aging of Nice (IRCAN) –“Aging and Diabetes” Team, Nice 06107, France; 3 INSERM U1081, IRCAN, Nice, France; 4 CNRS UMR7284, IRCAN, Nice, France; 5 Laboratory of Hereditary Diseases of Metabolism, East Center for Biology and Pathology, Civilian Hospices, Lyon, France

**Keywords:** Oligosaccharidoses, Mass spectrometry, MALDI-TOF/TOF

## Abstract

**Background:**

Oligosaccharidoses, which belong to the lysosomal storage diseases, are inherited metabolic disorders due to the absence or the loss of function of one of the enzymes involved in the catabolic pathway of glycoproteins and indirectly of glycosphingolipids. This enzymatic deficiency typically results in the abnormal accumulation of uncompletely degraded oligosaccharides in the urine. Since the clinical features of many of these disorders are not specific for a single enzyme deficiency, unambiguous screening is critical to limit the number of costly enzyme assays which otherwise must be performed.

**Methods:**

Here we provide evidence for the advantages of using a MALDI-TOF/TOF (matrix-assisted laser desorption ionization time-of-flight) mass spectrometric (MS) method for screening oligosaccharidoses. Urine samples from previously diagnosed patients or from unaffected subjects were randomly divided into a training set and a blind testing set. Samples were directly analyzed without prior treatment.

**Results:**

The characteristic MS and MS/MS molecular profiles obtained allowed us to identify fucosidosis, aspartylglucosaminuria, GM1 gangliosidosis, Sandhoff disease, α-mannosidosis, sialidosis and mucolipidoses type II and III.

**Conclusions:**

This method, which is easily run in less than 30 minutes, is performed in a single step, and is sensitive and specific. Invaluable for clinical chemistry purposes this MALDI-TOF/TOF mass spectrometry procedure is semi-automatizable and suitable for the urinary screening of oligosacharidoses.

## Introduction

Oligosaccharidoses, also called glycoproteinoses, are inherited metabolic diseases corresponding to a subset of lysosomal storage disorders resulting in deficient activity of one of the lysosomal hydrolases involved in the degradation of oligosaccharide components of glycoproteins. The glycosidic groups can be either N-linked or O-linked. These diseases are characterized by the abnormal accumulation of intermediate oligosaccharides in tissues and body fluids and by their excretion in the urine [1]. The metabolism of glycosphingolipids and glycosaminoglycans can also be impaired and lead to accumulation of these macromolecules. Oligosaccharidoses involve mainly the catabolic pathways for N-linked-glycoprotein breakdown that are bidirectional with sequential cleavages from both the reducing end of the oligosaccharide released after proteolysis, and from the non-reducing end (Additional file 1: Figure S1).

Oligosaccharidoses form a group of metabolic disorders [2] including fucosidosis (α-L-fucosidase deficiency), aspartylglucosaminuria (N-aspartyl-β-glucosaminidase deficiency), sialidosis (formerly called mucolipidosis type I, α-D-neuraminidase deficiency), GM1 gangliosidosis (β-D-galactosidase deficiency), Sandhoff disease also called GM2 gangliosidosis variant O (N-acetyl-β-D-hexosaminidase deficiency), α-mannosidosis (α-D-mannosidase deficiency) and β-mannosidosis (β-D-mannosidase deficiency). Oligosaccharidoses also include mucolipidoses type II and III (ML II and III), resulting from the deficiency of N-acetylglucosaminyl-1-phosphotransferase. This enzyme is required to generate a mannose-6-phosphate residue, the absence of which leads to incorrect addressing of hydrolases to the lysosomes [2]. All these diseases are characterized by the urinary accumulation of specific oligosaccharides (see Table 1) [3]. Note that abnormal oligosaccharides are also excreted in galactosialidosis due to neuraminidase and β-galactosidase deficiencies resulting from a primary lack in a third lysosomal protein, the bifunctional protein Protective Protein/Cathepsin A [2].

**Table 1:** Urine samples used for MALDI-TOF/TOF analysis of oligosaccharides randomly divided into a training set and a blind validation set

			**Number of samples**		
**Disease**	**Enzyme deficiency**	**Urinary oligosaccharide (OS) detected by TLC**	**Training set**	**Validation set**	**Total of samples**
**Fucosidosis**	α-L-Fucosidase	+++ (Fucosyl OS)	3	2	5
**Aspartylglucosaminuria**	N-Aspartyl-β-glucosaminidase	+++ (Glycoasparaginyl OS)	2	1	3
**Sialidosis**	α-D-Neuraminidase	+++ (Sialyl OS)	4	4	8
**GM1 gangliosidosis**	β-D-galactosidase	+++ (Galactosyl OS)	5	7	12
**Sandhoff disease**	N-acetyl-β-D-hexasominidase A and B	± (GlcNAc OS)	4	2	6
**α-mannosidosis**	α-D-mannosidase	+++ (Mannosyl OS)	7	4	11
**Mucolipidose type II**	N-Acetylglusaminyl 1-Phospho-transferase	ND (Sialyl OS)	6	1	7
**Mucolipidose type III**	N-Acetylglusaminyl 1-Phospho-transferase	ND (Sialyl OS)	4	2	6
**Unaffected (control)**	none	ND	6	30	36
		Total	41	53	94

Number of urine samples from patients unaffected or previously diagnosed to be affected with oligosaccharidose (TLC: Thin Layer Chromatography revealed using sulfuric orcinol, OS: oligosaccharides, GlcNAc: N-acetylglucosamine, +++: intensive spots, ±: weak spots, ND: not detectable).

The clinical signs of the different oligosaccharidoses are variable from one disorder to another, but also in terms of the severity among patients suffering from the same disease. However, oligosaccharidoses usually share some common clinical features including facial dysmorphism and progressive mental retardation [4]. Oligosaccharidoses can occur from early infant to adult age, and even at the prenatal stage.

Like for other lysosomal storage disorders (mainly mucopolysaccharidoses and sphingolipidoses), several trials have been undertaken for specific treatments [5]. Since oligosaccharidoses are rare diseases in humans, animal models have been developed for the validation of different therapeutic approaches, such as bone marrow transplantation (BMT), enzyme replacement therapy (ERT), substrate reduction therapy or cell transplant therapy [6-11]. More recently, gene therapy and pharmacological chaperones for enzyme enhancement have been tested [12,13]. Nonspecific BMT has been conducted in patients affected with aspartylglucosaminuria [14-16], fucosidosis [14,17], sialidosis [18], alpha-mannosidosis [14,19,20] and GM1 gangliosidosis [21], with usually incomplete recovery of the disease. Enzyme enhancement therapies have also recently been considered in GM1 and GM2 patients [22-24].

Since clinical features in many of these disorders are not characteristic for a single enzyme deficiency, effective screening is critical to limit the number of enzyme assays which otherwise should be performed. Current screening of oligosaccharidoses is routinely achieved on urine of suspected patients by profiling oligosaccharides and glycopeptides by thin layer chromatography (TLC) on silica gel and by revealing them using sulfuric orcinol [25]. Note that sialic acid-specific resorcinol staining can be used in addition to sulfuric orcinol staining to improve sialic residue detection. Testing oligosaccharidoses in urine is a non-invasive procedure which is useful especially with newborn patients. However, TLC analysis does not permit identification of the accumulated metabolites. Indeed, the abnormal presence of spots with a low retention factor is suggestive of an oligosaccharidosis. The diagnosis of the disorder can require a second TLC to confirm superimposition of the considered pattern with that from a patient affected with the incriminated oligosaccharidosis. Although TLC is technically simple it is time-consuming.

Improvement of the diagnostic test requires the development of more efficient and specific analytical methods. Several approaches have been tested including high performance liquid chromatography (HPLC) [26-28], but these have not been widely introduced in clinical chemistry laboratories. Over the last years, mass spectrometry (MS) has become the tool of choice for selective screening of inherited metabolic diseases and has made a major impact on detection strategies [29]. Different MS approaches have been described for oligosaccharidosis screening. However, to the best of our knowledge, these methods necessitate prior treatment of the urinary samples with derivatization such as permethylation or PMP (3-methyl-1-phenyl-2-pyrazolin-5-one) processes usually combined with HPLC or GC and confirmed by enzymatic digestion [3,30-32]. Although derivatization procedures bring improvements in sensitivity, these methods add procedures with possible sample deterioration and contamination, thereby potentially decreasing their benefits.

To improve the diagnosis of oligosaccharidoses, we propose here a direct method using matrix-assisted laser desorption/ionization (MALDI) and tandem mass spectrometry to obtain a MS/MS signature. This semi-automatizable approach is sensitive, specific and can be applied to the urinary screening of oligosaccharidoses.

## Materials and methods

### Patient samples

Urine samples were obtained from patients suspected of oligosacharidoses, and were stored at -20°C until analysis. Some samples were from patients affected with very rare oligosaccharide disorders, and were collected at the time of diagnosis some 30 years ago. Suspected patients, aged in majority between 1 month to 13 years with some up to 60 years, were suffering from mild or severe phenotypes with clinical signs most frequently including facial dysmorphism, dysostosis multiplex, hepatosplenomegaly, progressive neurodegeneration, deafness and visual impairment.

Ninety-four urine samples were analyzed. Fifty-eight were obtained from patients affected with various oligosaccharidoses. The diagnosis was ascertained in all cases on the basis of an abnormal characteristic TLC oligosaccharide pattern revealed using sulfuric orcinol [25] and of the demonstration of the specific enzymatic deficiency in serum and/or leukocytes and/or cultured skin fibroblasts (Laboratory of Hereditary Diseases of Metabolism, East Center for Biology and Pathology, Civilian Hospices, Lyon, France (57 cases), Biochemistry Laboratory, University Hospital, Nice, France (1 fucosidosis case)). Other urine samples from unaffected subjects (aged-matched to pediatric patients) were from the two laboratories. For mass spectrometry, samples were analyzed in two different sets. The first set was used for identification and characterization of the various MS/MS patterns; the second one was blindly analyzed for validation of the method (see Table 1).

### Materials

DHB (2,5-dihydroxybenzoic acid) and 3-AQ (3-aminoquinoline) matrices were purchased from Sigma-Aldrich (St. Louis, MO). All other chemicals were obtained from Merck (Darmstadt, Germany).

### Sample preparation

After thawing in an ice-bath, the urine samples were centrifuged for 5 min at 25.000 g at 4°C and the supernatants were diluted 1:3 in ultra-pure water. 0.5 μl were spotted on a MALDI 384 target plate ground steel with 2 μl of 3-AQ (15 g/l with 70% (v/v) of methanol, 1% (v/v) of NaCl 0.9% (w/v) and 0.2% (v/v) trifluoroacetic acid, TFA) for positive ion mode analysis and with 2 μl of DHB (50 g/l with 50% (v/v) of acetonitrile) for negative ion mode analysis. These preparations were done in triplicate and dried quickly with a hair-dryer.

### Mass spectrometric analysis

All measurements were recorded on an Ultraflex III MALDI-TOF/TOF (BrukerDaltonics, Bremen, Germany). External calibration was performed by spotting 0.5 μL peptide calibration standard II (BrukerDaltonics). All mass spectra were generated by summing 1,000 laser shots for reflectron ion mode, and 1,000 laser shots for the parent mass and at least 1,000 laser shots for the fragments in the lift mode. Laser power was adjusted between 15 and 30% of its maximal intensity, using a 200-Hz smartbeam laser. MS spectra were acquired in the reflectron positive ion mode within a mass range from 300 to 2,000 Da and in the reflectron negative ion mode within a mass range from 700 to 2,100 Da. Reflectron ion mode was chosen to obtain high detection sensitivity and resolution. Mass accuracy was about 14 ppm for 1347 Da. The FlexAnalysis version 3.0 and updated 3.4 provided by the manufacturer were applied for data processing. The GlycoWorkbench version 2.1 was used for the process of structure determination from mass spectrometry data and to draw glycan structures.

## Results

### Set up of the screening method

Our overall aim was to develop an accurate, simple and rapid method for the urinary screening of oligosaccharidoses using MALDI-TOF/TOF mass spectrometry. To characterize the MS and MS/MS profiles of a particular disease and to identify the accumulated oligosaccharides, we used a training set of urine samples from patients affected with different oligosaccharidoses (Table 1).

To set up the screening method, we took advantage of the relative tolerance for salt of MALDI mass spectrometry to directly analyze urine samples without prior treatment. As neutral glycans are able to ionize by capture of one sodium ion, we analyzed our samples in the positive ion mode meaning that the pseudomolecular ions detected correspond to [M + Na]^+^[33]. Note that in a salty medium, such as urine, oligosaccharides form adducts with sodium, but also with potassium resulting in a weaker supplemental pseudomolecular ion, [M + K]^+^. The latter is detected by a characteristic double peak separated by a 16 atomic mass unit (amu) difference, which corresponds to a potassium mass minus a sodium mass. Therefore, sodium salt was added to the sample matrix to favor sodium adducts. We also worked in the negative ion mode to detect sialylated glycans since the major form of sialic acid residue in the acidic urine pH is negatively charged. In this mode, the pseudomolecular ions observed correspond to molecular ions that have lost one proton, [M-H]^−^.

DHB and 3-AQ matrices are commonly used for glycan analysis by MALDI mass spectrometry [34-37]. We tested both matrices in positive and negative ion modes to optimize sample-matrix preparation to detect oligosaccharides without purification and derivatization procedures in urine samples. We adjusted our protocol based on a previous study for which we had to detect glycolipids (sialylated gangliosides and globosides) [38]. Both matrices allowed ionization in each mode. However, we obtained better signal intensity and fragmentation in the positive ion mode with 3-AQ matrix and in the negative ion mode with DHB matrix. Thus, the protocol has consisted in preparing each urinary sample in triplicate with 3AQ and DHB matrices. We recorded and analyzed MS data in reflectron positive and negative ion modes, and achieved the MS/MS analyses on intensive peaks for identification.

The MS/MS data do not provide absolute structure information for isomers such as *N*-acetylgalactosamine (GalNAc) or *N*-acetylglucosamine (GlcNAc), which is identified as N-acetylhexosamine (HexNAc). This is also the case for glucose, mannose and galactose, which are identified as hexoses. Predicted assignment of these oligosaccharide structures has been represented on MS spectra.

### Analysis of the training set

**Figure 1:**
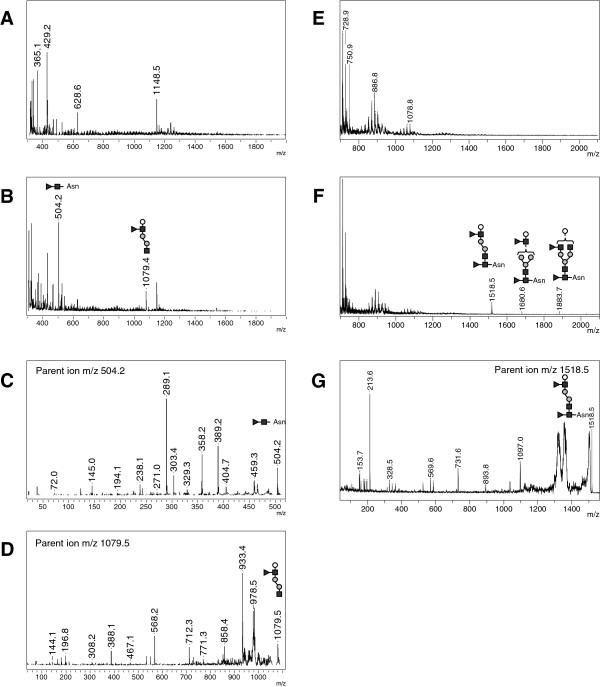
**Reflectron MALDI-TOF and MALDI TOF/TOF analysis of control and fucosidosis urines – Representative positive-ion (A) and negative-ion (E) MS spectra of control urine.** Representative positive-ion **(B)** and negative-ion **(F)** MS spectra of urine from fucosidosis affected patients with positive-ion MS/MS spectra of *m/z* 504.2 and 1079.5 **(C-D)** and negative-ion MS/MS spectrum of *m/z* 1518.6 **(G)**. Fucose (black triangle), N-acetylglucosamine (black squares), Mannose (grey circles), Galactose (open circles), Sialic acid (grey diamond), Asn (asparagine).

We first analyzed urines from subjects not suffering from oligosaccharidosis (controls). In positive ion mode, we always observed three intensive peaks from pseudomolecular ions at *m/z* 429.2, 628.6 and 1148.5 as shown on a representative MALDI-TOF MS spectrum (Figure 1A). We performed a MALDI-TOF/TOF (MS/MS) analysis for each of these ions with the goal to identify these compounds. However, no chemical composition could be related (data not shown). In negative ion mode, we constantly found peaks in the low molecular mass region *m/z* 700 to 1100 including pseudomolecular ions at *m/z* 728.9, 750.9, 886.8 and 1078.8, but no oligosaccharides could be identified (Figure 1E).

Analysis of urine from three patients affected with fucosidosis revealed a major pseudomolecular ion at *m/z* 504.2 and a second less intense one at *m/z* 1079.4 (Figure 1B), for which we were able to deduce the chemical composition. Carbohydrate fragmentation generates several different types of cleavage, and cationization can occur on different atoms [33,39,40]. The MALDI-TOF/TOF analysis of the parent ion at *m/z* 504.2 revealed a characteristic fragmentation with the more intense fragment ions at *m/z* 389.2, 358.2 and 289.1 (Figure 1C), corresponding respectively to a loss of an asparaginyl residue, a loss of a fucosyl residue and to a fragmentation inside the HexNAc cyclic form as previously described ([39] Additional file 1: Figure S2). Thus, the parent ion at *m/z* 504.2 corresponds to the [M + Na]^+^ ion of the Fuc-HexNAc-Asn (fucosyl-GlcNAc-Asparagine) oligosaccharide excreted in excess in urine of patients suffering from fucosidosis. Similarly, the MS/MS spectrum for the *m/z* 1079.5 parent ion revealed several specific fragments, among which the most intense one at *m/z* 933.4 corresponding to a loss of a fucosyl residue (Figure 1D). In negative-ion MS spectrum, we observed a peak at *m/z* 1518.5 with two weaker ones at *m/z* 1680.6 and 1883.7 for which we were able to deduce the chemical composition (Figure 1F), notably with the MS/MS analysis for the parent ion at *m/z* 1518.5 (Figure 1G). These oligosaccharides do not contain sialic acid, however they are detectable in negative mode thanks to the carboxyl group of asparagine.

**Figure 2:**
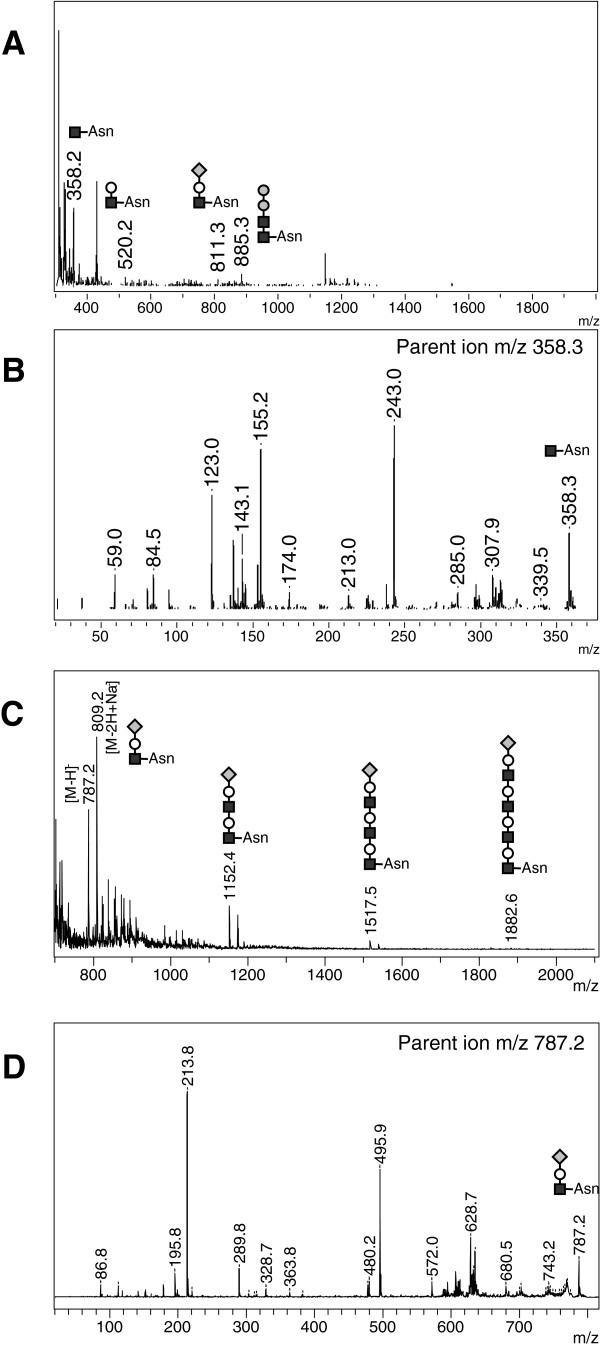
**Reflectron MALDI-TOF and MALDI TOF/TOF analysis of urine from aspartylglucosaminuria affected patient – representative positive-ion (A) and negative-ion (C) MS spectra with positive-ion MALDI MS/MS spectrum of *m/z *358.3 (B) and negative-ion MS/MS spectrum of *m/z *787.2 (D).** N-acetylglucosamine (black squares), Mannose (grey circles), Galactose (open circles), Sialic acid (grey diamond), Asn (asparagine).

Analysis of urine from two patients affected with aspartylglucosaminuria revealed a pseudomolecular ion at *m/z* 358.2 (Figure 2B). The MALDI-TOF/TOF analysis of the *m/z* 358.2 parent ion revealed a characteristic fragmentation with the more intense fragment ions at *m/z* 155.2 and 243.0 (Figure 2B), reflecting respectively a loss of an HexNAc residue, and the fragmentation of an asparaginyl residue as described above. This HexNAc-Asn compound predicted to be GlcNAc-Asn is known as the major stored compound in this disease (Figure 2A). We were also able to reproducibly detect lower intensity peaks at *m/z* 520.2, 811.3 and 885.3, which are expected to be glycoasparagine compounds as shown in Figure 2A. The negative-ion MS profile for urine from aspartylglucosaminuria affected patients showed two intensive peaks at *m/z* 787.2 and 809.2, corresponding respectively to [M-H]^-^ and [M-2H + Na]^-^ forms of the same compound, with some weaker peaks at *m/z* 1152.4, 1517.5 and 1882.6 (Figure 2C). The MS/MS analysis of the parent ion at *m/z* 787.2 gave characteristic fragments notably with the loss of a sialic residue identified at *m/z* 495.9 and the sialic residue at *m/z* 289.8 (Figure 2D).

**Figure 3:**
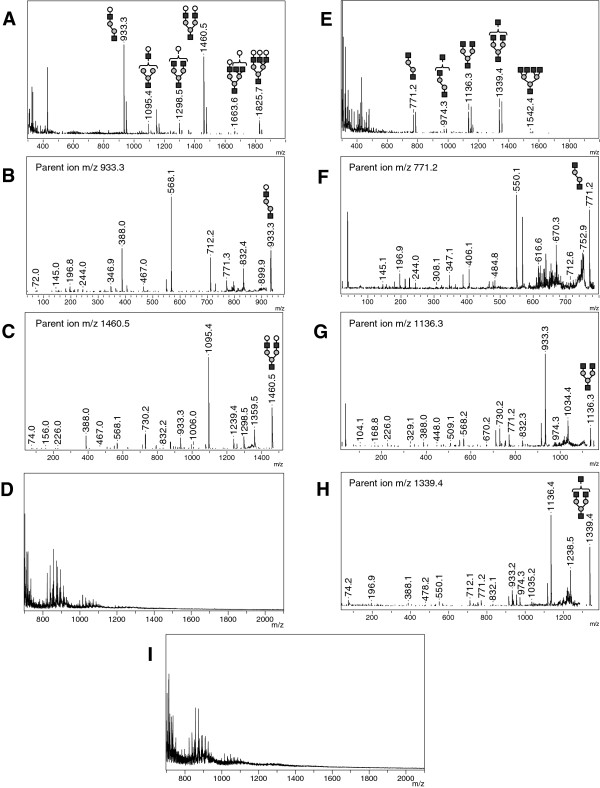
**Reflectron MALDI-TOF and MALDI TOF/TOF analysis of urine from gangliosidosis affected patients – Representative positive-ion MS spectra of GM1 (A) and Sandhoff (E) patients with positive-ion MS/MS spectra of respective intensive peaks (B-C, F-H).** Representative negative-ion MS spectra of GM1 **(D)** and Sandhoff **(I)** patients.N-acetylglucosamine (black squares), Mannose (grey circles), Galactose (open circles).

Analysis of urine from five GM1 gangliosidosis patients revealed two major pseudomolecular ions found at *m/z* 933.3 and 1460.5 in addition to lower intensity ions at *m/z* 1095.4, 1298.5, 1663.6 and 1825.7 (Figure 3A). All these peaks are separated either by a loss of 162 or 203 amu corresponding respectively to the loss of a hexose or an N-acetylhexosamine residue (Figure 3A). Morever, the MS/MS analysis on the more intense parent ions at *m/z* 933.3 (Figure 3B) and 1460.5 (Figure 3C) shows with confidence the reproducible and characteristic fragmentation profile of the major glycocompounds accumulated in urine from GM1 gangliosidosis patients. In negative ion mode, no characteristic peak was observed on MS spectrum (Figure 3D).

Analysis of urine from four patients affected with Sandhoff disease revealed characteristic pseudomolecular ions at *m/z* 771.2, 1136.3 and 1339.4 with lower intensity ions at *m/z* 974.3 and 1542.4. All these peaks are separated from each other by an amu difference corresponding to a hexose or a HexNAc residue (Figure 3E). We were able to perform MS/MS on the three more intense peaks at *m/z* 771.2 (Figure 3F), 1136.3 (Figure 3G) and 1339.4 (Figure 3H) which showed a signature with fragments typical for the identification of the Sandhoff disease. No characteristic peak was observed in negative-ion MS spectrum for this disorder (Figure 3I).

**Figure 4:**
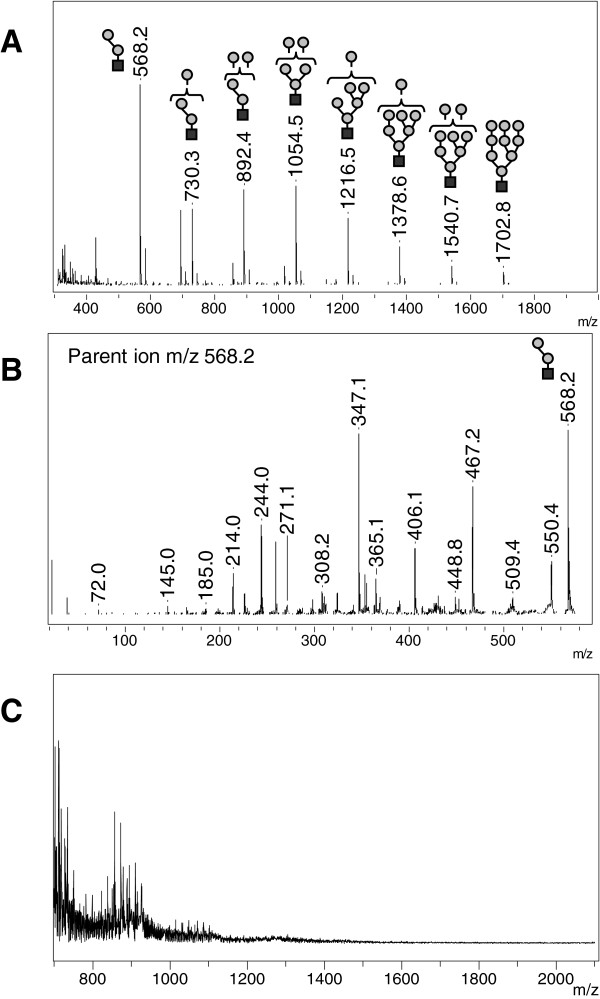
**Reflectron MALDI-TOF and MALDI TOF/TOF analysis of urine from α-mannosidosis affected patient – Representative positive-ion (A) and negative-ion (C) MS spectra with positive-ion MS/MS spectrum of m/z 568.2 (B).** Mannose (grey circles), N-acetylglucosamine (black squares).

Analysis of urine from seven patients affected with α-mannosidosis revealed a major peak at *m/z* 568.2 with additional peaks all separated by an amu difference corresponding to a hexose residue (Figure 4A). The MS/MS spectrum of the *m/z* 568.2 ion corresponds to a trisaccharide Hex-Hex-HexNAc with the specific peaks at *m/z* 365.1 agreeing with the loss of the HexNAc residue and at *m/z* 244.0 to the loss of two hexose residues (Figure 4B). This trisaccharide is probably Man-Man-GlcNac, which is the major compound described to be accumulated in α-mannosidosis patients (2). No characteristic peak was observed in negative-ion MS spectrum (Figure 4C).

**Figure 5:**
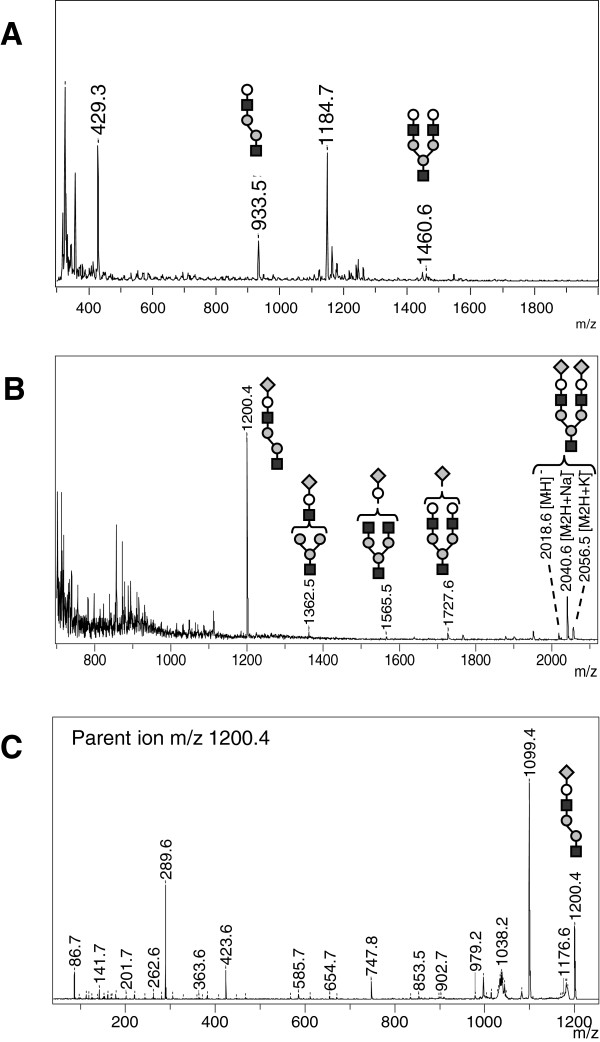
**Reflectron MALDI-TOF and MALDI TOF/TOF analysis of urine from patients suffering from sialidosis or MLII or MLIII – Representative positive-ion (A) and negative-ion (B) MS spectra with negative-ion MS/MS spectrum of m/z 1200.4 (C).** N-acetylglucosamine (black squares), Mannose (grey circles), Galactose (open circles), Sialic acid (grey diamond).

Analysis in positive ion mode of urine from four patients affected with sialidosis could reveal two weak characteristic peaks at *m/z* 933.5 and 1460.6 (Figure 5A). These two peaks are detected with higher intensity in urine from GM1 gangliosidosis affected patients (Figure 3A). Thus, the chemical structure of these ions corresponds to the same oligosaccharides as in GM1 gangliosidosis, *i.e*. oligosaccharides that have spontaneously lost their sialic acid residues during ionization/fragmentation in positive ion mode. Interestingly, in negative ion mode, the MS spectrum showed a pseudomolecular ion at *m/z* 1200.4 and less intense ions at *m/z* 1362.5, 1565.5, 1727.6 (Figure 5B). Among them, those at *m/z* 1200.4 and 1727.6 correspond to the oligosaccharides found in positive mode, with an additional sialic acid residue. The biantennary-disialylated oligosaccharide revealed three different peaks at *m/z* 2018.6, 2040.6 and 2056.5 corresponding respectively to [M-H]^-^, [M-2H + Na]^-^, and [M-2H + K]^-^ forms of the compound (Figure 5B). The MS/MS profile for the parent ion at *m/z* 1200.4, corresponding to the major sialylated compound, gave characteristic fragments with the sialic residue at *m/z* 289.6 (Figure 5C). Thus, the negative ion mode analysis allowed to detect sialylated oligosaccharides, which is not the case with the positive ion mode.

Finally, analysis of urine from six patients affected with MLII and four MLIII revealed MS and MS/MS spectra, in positive and negative ion modes, overall comparable from those obtained with urine from sialidosis affected patients (Figure 5). For some patients, biantennary-disialylated oligosaccharides were less detectable than for sialidosis. No urine from galactosialidosis affected patients could be analyzed, because of the rarity of the disease, but a similar pattern can be anticipated.

### Analysis of the validation set

The MS and MS/MS analyses performed on the training set of urine samples from patients known to suffer from various oligosaccharidoses enabled to identify characteristic accumulated oligosaccharides in urine and typical patterns that are useful for the diagnosis of new cases. The specificity of the method was then validated by blind analysis of a series of fifty-three samples including urine from control and oligosacharidosis affected patients (validation set, Table 1). Considering the rarity of these disorders, we have on purpose not taken into account the severity of the pathology when urine samples were randomly divided into the training and validation sets. The objective was to develop a specific assay allowing us to determine the predictive value of our protocol for oligosaccharidose screening in any circumstances.

Specific MS and MS/MS profiles of fucosidosis (2 cases), aspartylglucosaminuria (1 case), GM1 gangliosidosis (7 cases), Sandhoff disease (2 cases) and alpha-mannosidosis (4 cases) were identified from the above described characteristic patterns. Seven profiles corresponding to sialidosis/MLII/MLIII profiles were also recognized. After removal of patient anonymity, all the profiles observed were in perfect agreement with the previous diagnosis. None of the thirty control subjects showed any of the peaks characteristic for oligosaccharidosis.

In summary, analysis of ninety-four urine samples, with the training and the validation sets, made it possible to identify fifty-eight characteristic pathological patterns from fifty-eight urine samples previously diagnosed by TLC/enzymatic assays for different oligosaccharidoses.

## Discussion

The clinical diagnosis of oligosaccharidoses can be challenging because of the great variability in symptoms. The recent development of specific therapies for lysosomal storage disorders, and particularly for oligosaccharidoses, raises the medical request for oligosaccharidosis screening in order to offer the opportunity to the patient to benefit from a precise diagnosis and hence the appropriate treatment. To date, screening is commonly performed by TLC [25,27] in laboratories working in the field of inborn errors of metabolism. This time-consuming method permits to evoke a number of possibilities for the suspected oligosaccharidosis, but in most cases the precise diagnosis is subsequently confirmed by enzymatic analysis and in some cases by molecular biology. Efforts to improve the oligosaccharidosis screening have been achieved using mass spectrometry. For instance, the group of Michalski developed a strategy combining three different approaches with MALDI-TOF-MS and GC-MS after permethylation and enzymatic digestions to identify the glycocompounds accumulated in urine [30]. More recently, a method using PMP derivatization combined with LC-MS/MS has been adapted to screen oligosaccharidoses by selected reaction monitoring MS, and it revealed specific patterns corresponding to each disorder [32]. While our report was under review, Xia et al. also reported that MALDI-TOF-MS could be used for the diagnosis of lysosomal storage diseases after sequential passage over different columns and permethylation of urine samples [41]. These approaches used laborious and expensive pre-analytic treatment of urines mainly to detect sialylated oligosaccharides and glycoamino acids. Indeed, these chemical stabilization procedures are used to increase ionization efficiency of oligosaccharides and to prevent spontaneous loss of labile sialic acid residues during ionization and fragmentation.

The screening strategy that we present here with MALDI/TOF-TOF tandem mass spectrometry offers multiple advantages. These include the analysis in a short time on a very small amount of urine with an optimized and simple sample-matrix-preparation procedure and a high specificity and sensitivity of detection. In positive ion mode we are able to directly detect and identify complex oligosaccharides and glycoamino acids in urines from oligosaccharidosis affected patients without any sample preparation. Importantly, using the negative ion mode we revealed negatively charged sialylated oligosaccharides, which is not possible with the positive ion mode. However, the chemical structure of sialyl-oligosaccharides, with the presence of a carboxyl group at the anomeric carbon of sialic acid, easily explains the lack of signal of sialyl-oligosaccharides in positive mode, and the readily formed [M-H]^-^ ions in MS/MS negative mode. Note that we have tested matrices other than DHB, such as 9-aminoacridine or 1,5-diaminonaphtalene, without better reliable results in this mode (data not shown). Thus, only the negative ion mode is suitable to detect directly sialylated oligosaccharides.

Urine is known to contain some simple oligosaccharides but their excretion levels depend on various physiological circumstances [30]. Indeed, it has previously been reported in chemically derivated urine (e.g. permethylated urines) that some oligosaccharides can be detected by mass spectrometry [30,41]. In our study, we found in urines from healthy subjects, with a similar abundant pattern, a set of intensive peaks for which no chemical composition could be identified. To avoid misinterpretation, we have analyzed additional samples (n > 50), and we obtained the same kind of profile as previously found for the controls without oligosaccharides identified from the more intensive peaks. Therefore, some oligosaccharides might be present in the urines from unaffected subjects but in very low abundance compared to the one accumulated in pathological urines, and being therefore undetectable by our method without pre-analytical treatment.

The MALDI-TOF/TOF analysis presents a relative simplicity of spectral interpretation directly obtained from native urine oligosaccharides with singly charged ions. It provides a reliable oligosaccharide pattern, with a specific MS and MS/MS signature for each oligosaccharidosis resulting in a positive argument for the diagnosis of these disorders when present, and an argument for the exclusion of these disorders when absent. Indeed, it gives a structural analysis enabling the identification of the chemical composition (except for isomers as mentioned above) and of the structure of the accumulated oligosaccharides characteristic of each disease. This approach has a very high sensitivity and resolution permitting the detection of all the characteristic compounds for each disorder, independent of patient age and/or disease severity. Moreover, for analysis of pediatric patients the scant volume of the samples is a limitation for several screening procedures. Importantly, the protocol developed here stands out as it requires a minimal volume of urine (10 μl) and can be easily performed in less than 30 min without expensive sample preparation.

Precise quantification of the amount of accumulated oligosaccharides in each disease could be useful for the follow-up of the treatment and would require an internal standard, possibly deuterated. In this case, the results would require to be reported to the creatinine level, which is a variable parameter measuring the urine concentration often used as a correcting factor in many analytical methods involving urine. In our study, we performed a run of samples with creatinine concentrations standardized to 1 nmol/l. We observed the same MS profile compared to results obtained without creatinine correction (data not shown).

Despite the relative low frequency of these diseases, we had the possibility to study between 3 to 12 samples for each oligosaccharidosis. Each sample was analyzed in triplicate on three different days, in order to estimate the intra- and the inter-assay reproducibility, but no difference was found. After identification of the profiles on the samples of the training set, the method was validated by blindly analyzing a second set of samples, which renders our study particularly original and powerful. No false negative results were noticed. Thus, the reproducibility and specificity of our approach allowed us to characterize with confidence eight different oligosaccharidoses with urinary accumulation of uncompletely degraded oligosaccharides. The same oligosaccharide pattern (mainly sialyl-oligosaccharides) was observed in sialidoses, ML II and ML III. Therefore, we have re-analyzed all our samples from patients suffering from sialidosis, ML II and ML III after the training and validation analyses. We compared their MS spectra in terms of peak intensity and we were able to rank them in ascending order MLIII < MLII < sialidosis (data not shown). ML II and ML III could not be detected by the TLC oligosaccharide method using sulfuric orcinol staining. These diseases were distinguished and diagnosed by additional enzymatic tests, which are always requested for assessing the correct diagnosis, whatever the oligosaccharide analysis method used. Note that abnormal sialyl-oligosaccharides would have been probably revealed with chlorhydric resorcinol staining of TLC plates. Therefore, our method brings more information than the prior TLC method using orcinol.

In conclusion, the strategy we developed here with the MALDI-TOF/TOF mass spectrometry for the urinary screening and identification of oligosaccharidoses is accurate and performed in a single rapid step. Hence, this screening method of these metabolic disorders could be easily adapted in clinical chemistry laboratories equipped with a MALDI-TOF/TOF mass spectrometric apparatus. Interestingly, this method could further be adjusted to other kinds of samples (amniotic fluid, ascites and maybe serum or cultured cells) and to other lysosomal storage diseases (glycosphingolipidoses, mucopolysaccharidoses).

## Competing interests

The authors declare that they have no competing interests.

## Authors’ contributions

CH and RM conceived and designed the experiments. LB performed the experiments. LB, CH, RM and MP analyzed the data. MP and CC contributed to sample collection. CH, MP, LB, CC and EVO contributed to the writing of the paper. All authors read and approved the final manuscript.

## Supplementary Material

Additional file 1: Figure S1 Bidirectional catabolic pathway of N-linked oligosaccharides and associated diseases. **Figure S2.** Different posibilities of cationization with sodium for the fucosyl-GlcNac-asparagine residue leading to different fragmentations of the parent ion at m/z 504 in positive mode deduced with HighChem Mass Frontier 5.1 logiciel. Click here for file
