# Supplementary material for: Fast urinary screening of oligosaccharidoses by MALDI-TOF/TOF mass spectrometry
Source: Orphanet J Rare Dis. 2014 Feb 6;9:19. doi: 10.1186/1750-1172-9-19 (PMC3922009; doi:10.1186/1750-1172-9-19)
Supplement: Additional file 1: Figure S1 — Bidirectional catabolic pathway of N-linked oligosaccharides and associated diseases. Figure S2. Different posibilities of cationization with sodium for the fucosyl-GlcNac-asparagine residue leading to different fragmentations of the parent ion at m/z 504 in positive mode deduced with HighChem Mass Frontier 5.1 logiciel. [file 1750-1172-9-19-S1.ppt]

## Slide 1
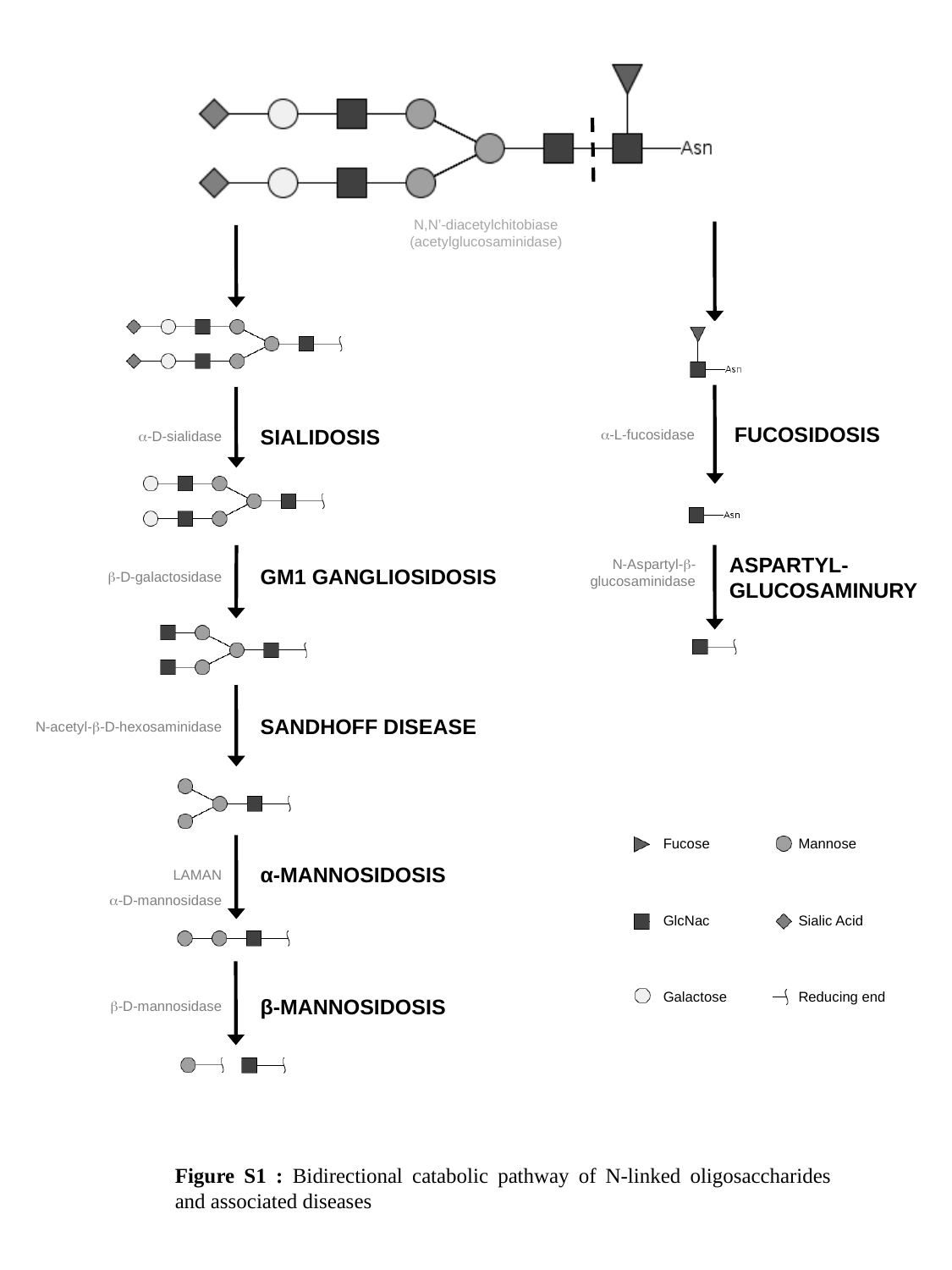

N,N’-diacetylchitobiase
(acetylglucosaminidase)
FUCOSIDOSIS
SIALIDOSIS
α-L-fucosidase
α-D-sialidase
ASPARTYL-GLUCOSAMINURY
N-Aspartyl-β-glucosaminidase
GM1 GANGLIOSIDOSIS
β-D-galactosidase
SANDHOFF DISEASE
N-acetyl-β-D-hexosaminidase
Fucose
Mannose
α-MANNOSIDOSIS
LAMAN
α-D-mannosidase
Sialic Acid
GlcNac
Galactose
Reducing end
β-MANNOSIDOSIS
β-D-mannosidase
Figure S1 : Bidirectional catabolic pathway of N-linked oligosaccharides and associated diseases

## Slide 2
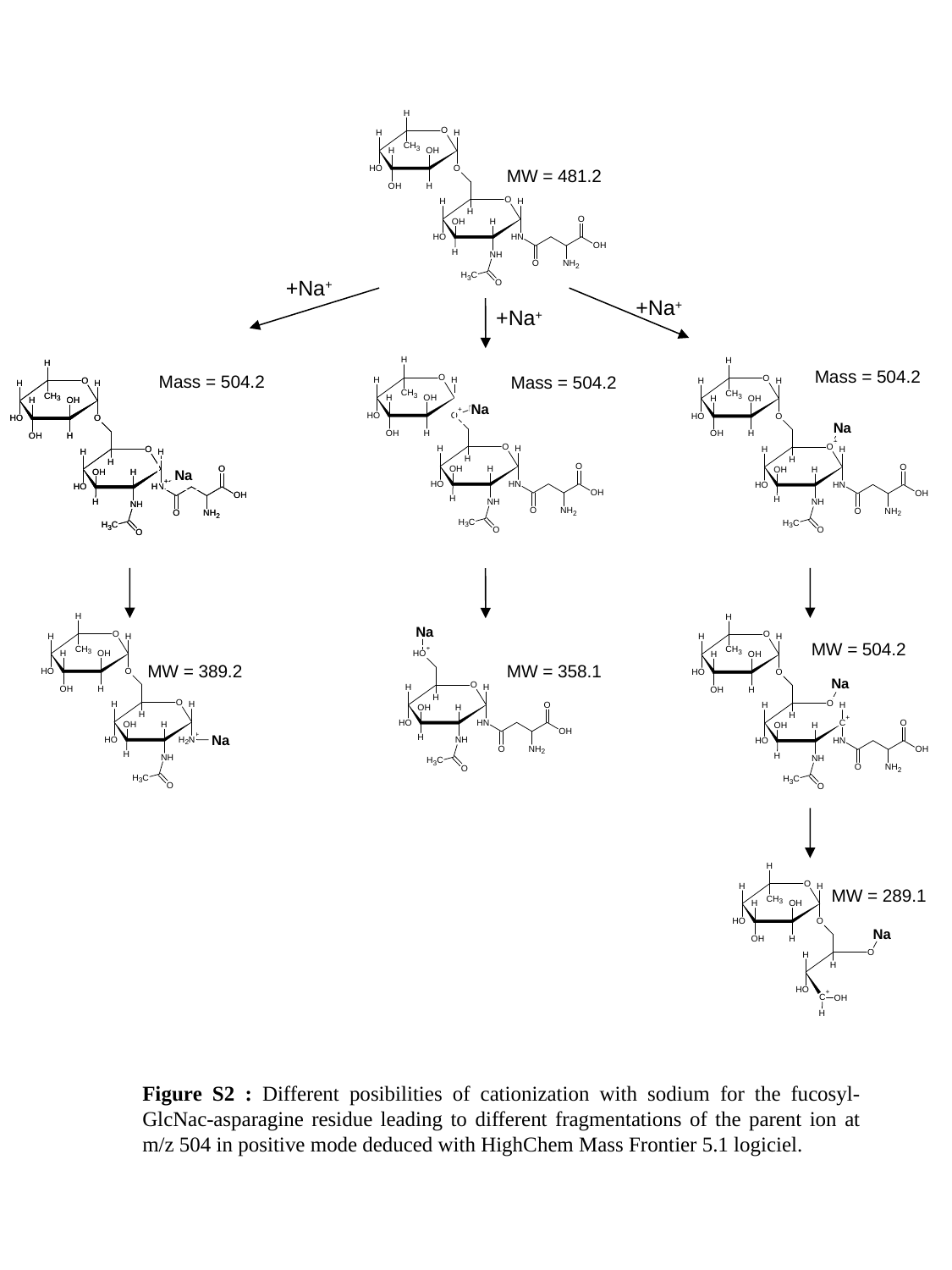

MW = 481.2
+Na+
+Na+
+Na+
Na
Mass = 504.2
Mass = 504.2
Mass = 504.2
Na
Na
Na
Na
Na
MW = 504.2
MW = 389.2
MW = 358.1
Na
MW = 289.1
Figure S2 : Different posibilities of cationization with sodium for the fucosyl-GlcNac-asparagine residue leading to different fragmentations of the parent ion at m/z 504 in positive mode deduced with HighChem Mass Frontier 5.1 logiciel.
